# DNA repair capacity as a possible biomarker of breast cancer risk in female *BRCA1* mutation carriers

**DOI:** 10.1038/sj.bjc.6603528

**Published:** 2007-01-09

**Authors:** J Kotsopoulos, Z Chen, K A Vallis, A Poll, P Ainsworth, S A Narod

**Affiliations:** 1 Centre for Research in Women's Health, Women's College Hospital, 790 Bay Street, Room 750, 7th Floor, Toronto, Ontario, Canada M5G 1N8; 2 Department of Nutritional Sciences, Faculty of Medicine, University of Toronto, FitzGerald Building, 150 College Street, Toronto, Ontario, Canada M5S 3E2; 3 Department of Radiation Oncology, Princess Margaret Hospital, 610 University Ave, Toronto, Ontario, Canada M5G 2M9; 4 London Health Sciences Center, Molecular Diagnostic Laboratory, Room E3-304, Westminster Tower, 800 Commissioner's Road East, London, Ontario, Canada N6A 4G5

**Keywords:** BRCA1, DNA repair, biomarkers, radiosensitivity

## Abstract

The *BRCA1* gene product helps to maintain genomic integrity through its participation in the cellular response to DNA damage: specifically, the repair of double-stranded DNA breaks. An impaired cellular response to DNA damage is a plausible mechanism whereby *BRCA1* mutation carriers are at increased risk of breast cancer. Hence, an individual's capacity to repair DNA may serve as a useful biomarker of breast cancer risk. The overall aim of the current study was to identify a biomarker of DNA repair capacity that could distinguish between *BRCA1* mutation carriers and non-carriers. DNA repair capacity was assessed using three validated assays: the single-cell alkaline gel electrophoresis (comet) assay, the micronucleus test, and the enumeration of *γ*-H2AX nuclear foci. DNA repair capacity of peripheral blood lymphocytes from 25 cancer-free female heterozygous *BRCA1* mutation carriers and 25 non-carrier controls was assessed at baseline and following cell exposure to *γ* – irradiation (2 Gy). We found no significant differences in the mean tail moment, in the number of micronuclei or in the number of *γ*-H2AX nuclear foci between the carriers and non-carriers at baseline, and following *γ*-irradiation. These data suggest that these assays are not likely to be useful in the identification of women at a high risk for breast cancer.

The inheritance of a deleterious mutation in the breast cancer susceptibility gene, *BRCA1*, has been associated with a lifetime risk of breast cancer of between 45 and 87% (Ford *et al*, 1998; Antoniou *et al*, 2003). Genetic, reproductive, and environmental factors have all been suggested to influence breast cancer risk in *BRCA1* mutation carriers (reviewed in Narod and Offit (2005)). The use of breast cancer as an endpoint to evaluate the protective role of potential modifying factors is not always feasible. Thus, there is a need to ascertain biomarkers of cancer susceptibility in these women. In turn, the identification of a valid biomarker of breast cancer risk would allow us to identify mutation carriers and help improve our ability to target, and to evaluate the effect of both dietary and lifestyle alterations, as well as, medical diagnostic and therapeutic interventions on breast cancer risk.

The BRCA1 protein plays a vital role in maintaining genomic stability because of its key role in the repair of double-stranded DNA breaks by homologous recombination (reviewed in Welcsh *et al* (2000)). If double-stranded DNA breaks are left unrepaired, or are repaired inaccurately, aberrant chromosome breaks, deletions, and translocations may accumulate. Thus, an impaired cellular response to DNA damage appears to be a plausible mechanism whereby *BRCA1* mutation carriers are at an increased risk of breast cancer (Scott, 2004). Hence, the evaluation of an individual's capacity to repair DNA may serve as a biomarker of breast cancer risk in carriers of these mutations.

Two previous studies have shown higher levels of chromosomal aberrations and chromosomal breaks among women with a *BRCA1* mutation compared with non-carrier controls (Kowalska *et al*, 2005; Kote-Jarai *et al*, 2006); however, these cytogenetic assays are laborious and expensive. The aim of the current paper was to elucidate a relatively simple and inexpensive biomarker of breast cancer susceptibility. We compared the DNA repair capacity of *BRCA1* mutation carriers and non-carriers using three previously validated assays, the single-cell alkaline gel electrophoresis (comet) assay, the micronucleus test, and *γ*-H2AX staining, to determine which, if any of these tests, would be able to predict the presence of an inherited *BRCA1* mutation.

## MATERIALS AND METHODS

### Subjects and study design

Eligible subjects were healthy females with no prior history of breast or other cancers and were between the ages of 20–60 years. We included 25 healthy *BRCA1* mutation carriers and 25 healthy mutation-negative females as controls. The controls were women from the same families as the cases; that is a mutation had been identified in the family but they did not carry the family mutation. Potential subjects were identified from two participating centres: the Centre for Research in Women's Health, Toronto, Ontario and the London Health Sciences Centre, London, Ontario. Women were invited to participate in the study by letter. Women were excluded if they were pregnant or were suffering from a serious illness. The majority of women who were approached agreed to participate. The reasons given for declining our invitation included (1) travel time to our clinic in downtown Toronto (many individuals live outside the Greater Toronto Area), (2) dealing with other family matters, (3) not interested, or (4) lost to follow-up.

### Data collection

Participants completed three questionnaires before their visit to the clinic. These included a diet history questionnaire (a food frequency questionnaire that was developed by staff at the Risk Factor Monitoring and Methods Branch at the National Cancer Institute and reflects Canadian food availability and food fortification practices) (Csizmadi *et al*, 2006), a ‘follow-up questionnaire for a study of breast and ovarian cancer in high-risk families' with questions directed at reproductive histories, prophylactic surgery, use of exogenous estrogens, and other lifestyle factors, and a shorter ‘research questionnaire for a study of genetic and non-genetic factors associated with breast cancer risk in high-risk women’ that asked questions regarding use of dietary supplements and physical activity.

At an on-site visit, biological samples were collected from all the eligible participants, and were processed and stored for further analyses. These included fasting blood samples, toenail clippings, and urine samples. Standardised procedures were used to obtain various anthropometric measurements (weight, waist, and hip circumference, height).

### Measurement of DNA repair capacity

The comet assay, micronucleus test and analysis of *γ*-H2AX nuclear foci were performed using lymphocytes extracted from freshly collected blood. The assays were performed in the laboratory of Dr Katherine Vallis at the Ontario Cancer Institute, Princess Margaret Hospital, Toronto, Ontario.

#### Lymphocyte isolation and culture

Five millilitres (ml) of blood were collected from female heterozygous *BRCA1* mutation carriers and non-carrier controls by venipuncture into a heparin tube (1 unit ml^−1^) and kept on ice before being added into 25 ml of ice-cold Rosewell Park Memorial Institute medium (RPMI) 1640 medium supplemented with 2% foetal bovine serum (FBS). Twenty ml of Ficoll was added to the bottom of the blood-RPMI cell culture media. After centrifugation at 1200 r.p.m. for 15 mins at 4°C, lymphocytes were collected from the layer between the RPMI medium and Ficoll.

#### Single cell gel electrophoresis (comet) assay

Lymphocytes were either not irradiated (controls) or treated with 2 Gy of *γ* irradiation (^137^Cs *γ* rays, dose rate of 1.07 Gy min^−1^) and placed on ice for no recovery or allowed to recover for 1 h at 37°C. The irradiated cells were then mixed with 100 *μ*l of 0.5% low melting agarose (melted and kept warm at 42°C in phosphate-buffered saline (PBS)) before being loaded onto the slides pre-coated with 1% of agarose.

The DNA damage and repair were determined by the alkaline comet assay as per standard protocols (Singh *et al*, 1988; Green *et al*, 1992). All procedures preceding electrophoresis were carried out on ice to prevent DNA repair except when samples were deliberately set aside to allow for repair. Data were analysed using a Zeiss fluorescence microscope with a × 20 objective and image-analysis software, Komet 5.0 (Kinetics Imaging Ltd., Liverpool, UK). A minimum of 50 cells per slide were analysed. The tail moment was calculated by the distance between the centre of mass of the tail and the centre of mass of the head multiplied by the percentage of DNA in the tail (Olive *et al*, 1990). An increase in the tail moment provides evidence of an increased number of cellular DNA strand breaks.

#### Micronucleus test

Lymphocytes were stimulated with phytohaemagglutinin (PHAP) (Sigma, 10 *μ*g ml^−1^ final) and were allowed to grow for 20 h in 10 ml of RPMI with 10% FBS, 37°C and 5% CO_2_. Twenty hours after treatment with PHAP, cells were not irradiated (controls) or irradiated with 2 Gy (^137^Cs *γ* rays, dose rate of 1.07 Gy min^−1^). Forty-four hours after PHAP stimulation, cytochalasin B (Sigma) was added to the cell culture (to a final concentration of 4.5 *μ*g ml^−1^) to inhibit cytokinesis (cytoplasmic division) without interfering with nuclear division. Thus, cytochalasin B-treated cells become binucleated at the first cell division. Sixty-eight hours after PHAP treatment, cells were collected by centrifugation, treated with hypotonic solution (70 mM KCl), fixed with methanol/acetic acid (9 : 1), and stained with 0.01% acridine orange (Sigma: Sigma-Aldrich Canada Ltd., Oakville, ON, Canada).

A fluorescent microscope was used to score micronuclei. The results were recorded as the number of micronuclei per 1000 binucleated cells. The micronuclei were scored as positive if they were distinguishable from the two main nuclei, if they were less than one third the size of the main nuclei, and if they had similar staining intensities to the main nuclei. Cells with irregularly shaped nuclei, more than two nuclei, and those with nuclei of different sizes in a single cell were not scored (Khan *et al*, 1998). The number of micronuclei per binucleated cell was scored to provide a measure of chromosome breakage (Fenech, 2000). A minimum of 200 cells per slide were scored.

#### Immunohistochemistry for *γ*-H2AX detection

Lymphocytes were not irradiated (controls) or *γ*-irradiated (2 Gy). After incubation at 37°C for 30 min and 3 h, cells were spun down onto microscope slides using a cytocentrifuge (Cytospin, Shandon; Thermo Fisher Scientific, Waltham, MA, USA) 7 min at 1200 r.p.m.). The cells were fixed by dipping the slides into 2% paraformaldehyde in PBS+0.5% Triton X-100, pH 8.2, 15 min at room temperature (Reitsema *et al*, 2004; Al Rashid *et al*, 2005). The primary antibody, anti-phospho-H2AX (mouse monoclonal clone JBW301, Upstate, Millipore Corp, Billerica, MA, USA’ – Note that Upstate has been taken over by Millipore) was 1 : 500 diluted in filtered 3% bovine serum albumin in PBS, and incubated overnight at 4°C. After the secondary antibody application, lymphocyte nucleus staining was performed by incubating cells with 0.1 *μ*g ml^−1^ diaminophenyl indole (DAPI) for 10 min at room temperature. Wide-field fluorescence images were captured using a Zeiss Axioskop microscope with the × 100 objective, filter sets for fluorescein isothiocyanate and DAPI, and a Retiga CCD camera (QIMAGING, British Columbia, Canada). Northern Eclipse (EMPIX, Mississauga, Canada) software was used to acquire 8-bit images from which at least 50 cells per slide were scored. One *γ*-H2AX focus has been shown to be equivalent to one DSB and is made up of 100's–1000's of *γ*-H2AX molecules (Pilch *et al*, 2003).

### Statistical analysis

#### Descriptives

The Student's *t*-test was used to compare normally distributed continuous variables between *BRCA1* mutation carriers and the non-carrier control subjects. The *χ*^2^ test was used to test for categorical differences.

#### Multivariate analysis

The primary objective of this study was to examine whether any of the three assays could distinguish between *BRCA1* mutation carriers and non-carriers. To do so, unadjusted and adjusted values were compared between the two groups of women. Differences in the mean DNA repair capacity between *BRCA1* mutation carriers and the non-carrier control subjects were compared using the Student's *t*-test. Multivariate linear regression analysis was performed on the three markers of DNA repair capacity treated as continuous dependent variables, and adjusting for the potential confounding effects of age (continuous), body mass index (kg m^−2^), current smoker (yes/no), total drinks of alcohol (continuous), total hours of physical activity per week (continuous), and total caloric intake (kcal) (Berwick and Vineis, 2000; Palli *et al*, 2003).

#### Percentage of radiosensitive women

For each of the three assays, we estimated the number of radiosensitive individuals. An arbitrary cut-off was based on the 90th percentile of the non-carrier population (i.e. assumes that 10% of the population is sensitive) (data not shown). The Fisher's exact test was used to test for differences in the percentage (%) of radiosensitive individuals between *BRCA1* mutation carriers and non-carriers.

All statistical tests were two-sided. A *P*-value of 0.05 was taken to be significant. All analyses were performed using the SPSS statistical package, version 12.0.1 for Windows.

## RESULTS

### Baseline characteristics of study subjects

Fifty women were enrolled in the current study, including 25 *BRCA1* mutation carriers and 25 non-carrier controls. *BRCA1* mutation carriers and controls were similar with respect to current age, age at menarche, age at first birth, body mass index, and oral contraceptive use (Table 1). The number of postmenopausal women was slightly higher among carrier women (15 *vs* 8, *P*=0.05), and a higher proportion of carriers were current users of hormone replacement therapy (16.0 *vs* 0% in carriers and non-carriers, respectively; *P*=0.04). Smoking status, energy intake per day, alcohol consumption, and the total hours of physical activity per week were similar for carriers and non-carriers (Table 1).

### Comet assay

Table 2 summarises the tail moments in the *BRCA1* mutation carriers and the non-carrier controls at baseline (before irradiation or 0 Gy), immediately following 2 Gy of *γ*-irradiation (no recovery time), and 1 h following 2 Gy of *γ*-irradiation. As shown in Table 2, there was no significant difference in the mean tail moment between the *BRCA1* mutation carriers and the controls at baseline (0 Gy), following 2 Gy of *γ*-irradiation (no recovery time), and following 2 Gy of *γ*-irradiation with 1 h of recovery (*P* ⩾ 0.70 for all). Adjustment for potential confounders of DNA repair capacity did not affect the results (see Table 2).

**Figure 1:**
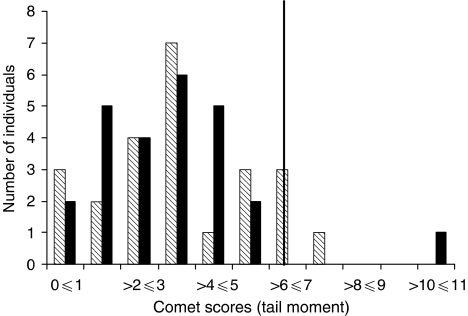
Distribution of radiation-induced mean tail moments in the entire study population. Shaded boxes represent women who are *BRCA1*^*WT*^ and the black boxes represent women who are *BRCA1*^*+/−*^. The solid vertical line represents the cutoff point between radiosensitive and non-sensitive individuals and was based on the 90^th^ percentile of the non-carrier population after 2 Gy of *γ*-irradiation and one hour of recovery time (cutoff=6.39).

Using the 90th percentile of the non-carrier population, we were able to classify the study subjects as sensitive or non-sensitive individuals based on this assay (Figure 1). After 2 Gy of *γ*-irradiation and 1 h of recovery, 12.0% of the women with a *BRCA1* mutation (three out of 25) and 8.3% of non-carrier controls (two out of 24) were judged to be radiosensitive. This suggests no elevated predisposition to sensitivity among mutation carriers based on the comet assay (Table 2).

### Micronucleus test

No significant differences were observed using the micronucleus test to quantify DNA repair capacity (Table 3). There was no significant differences in the mean number of MN per 1000 binucleated cells between the women with a *BRCA1* mutation compared with the non-carrier controls at baseline (before irradiation or 0 Gy), as well as immediately following 2 Gy of *γ*-irradiation (no recovery time) (*P*=0.12 and 0.82, respectively). Adjusting for potential confounders did not affect the results (Table 3).

**Figure 2:**
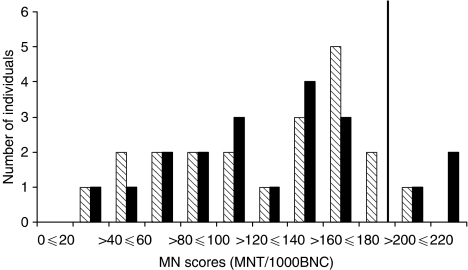
Distribution of radiation-induced micronucleus frequencies in the entire study population. Shaded boxes represent women who are *BRCA1*^*WT*^ and the black boxes represent women who are *BRCA1*^*+/−*^. The solid vertical line represents the cutoff point between radiosensitive and non-sensitive individuals and was based on the 90^th^ percentile of the non-carrier population after 2 Gy of *γ*-irradiation and no recovery time (cutoff=197.69).

Figure 2 illustrates the distribution of the MN per 1000 binucleated cells scores of the entire study population. Using the 90th percentile of the non-carrier controls as the cut-off point for radiosensitivity (cut-off=197.69 MN per 1000 binucleated cells), 15% (three out of 20) of the *BRCA1* mutation carriers were radiosensitive compared to 9.5% (two out of 21) of the non-carriers (data not shown). This difference was not significant (*P*=0.66).

### *γ*-H2AX staining

The number of *γ*-H2AX foci per cell were quantified at baseline (before irradiation or 0 Gy), at 30 min and at 3 h following 2 Gy of *γ*-irradiation (Table 3). There were no significant differences in the number of *γ*-H2AX foci per cell between *BRCA1* mutation carriers and non-carriers at any of the three time points (*P*⩾0.45 for all) (Table 4).

**Figure 3:**
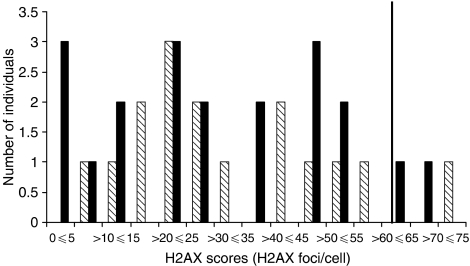
Distribution of radiation-induced *γ*-H2AX foci in the entire study population. Shaded boxes represent women who are *BRCA1*^*WT*^ and the black boxes represent women who are *BRCA1*^*+/−*^. The solid vertical line represents the cutoff point between radiosensitive and non-sensitive individuals and was based on the 90th percentile of the non-carrier population after 2 Gy of *γ*-irradiation and 3 h of recovery time (cutoff=62.01).

The distribution of the scores using *γ*-H2AX staining and the 90th percentile of the non-carriers using *γ*-H2AX staining are shown in Figure 3 (cut-off=62.01). Using this value, the percentage of sensitive *BRCA1* mutation carriers was not significantly different compared with the non-carriers (10.0% (two out of 19) *vs* 6.3% (one out of 16), *P*=1.00, respectively) (data not shown).

## DISCUSSION

The aim of the current study was to elucidate a simple, rapid, and inexpensive biomarker for use in future intervention studies of high-risk women. We examined the potential for the comet assay, micronucleus test, and *γ*-H2AX staining to discriminate between women with and without a *BRCA1* mutation. We found no difference in DNA repair capacity using any of these tests. These assays were chosen because they are rapid and simple, and they have successfully been employed to screen for radiation-sensitive individuals and individuals with cancer-prone syndromes (Rothfuss *et al*, 1998; Djuzenova *et al*, 1999; Kuhne *et al*, 2004). As a panel, the three tests measure both the immediate effect of radiation on DNA breakage, and DNA repair capacity up to 24 h post-irradiation. H2AX is a histone, which within seconds of damage to double-stranded DNA, becomes phosphorylated to yield *γ*-H2AX that form foci that flank regions of the double-stranded DNA breaks (Pilch *et al*, 2003). One *γ*-H2AX focus has been shown to be equivalent to one double-strand break (Sedelnikova *et al*, 2002) and contain hundreds to thousands of *γ*-H2AX molecules (Rogakou *et al*, 1998). *γ*-H2AX nuclear foci are counted using immunofluorescence. Detection of foci is used to evaluate the induction of radiation-induced double-stranded DNA breaks in lymphocytes (Petersen *et al*, 2001; Fernandez-Capetillo *et al*, 2003; Pilch *et al*, 2003; Rothkamm and Lobrich, 2003; Kuhne *et al*, 2004). The alkaline comet assay measures both acute DNA damage and rate of repair (Olive, 2002) and was measured immediately post-irradiation and 1 h thereafter. The micronucleus test is a variant of the chromosome aberration assay and measures the frequency of micronuclei in binucleated cells, as an estimate of chromosomal breakage (Wojewodzka *et al*, 1997; Rothfuss *et al*, 2000; Westphal *et al*, 2003). Micronucelei were measured before irradiation and 24 h after irradiation. We observed no significant differences in the mean values of DNA damage before *γ*-irradiation or following repair, or in the percentage of sensitive individuals, for any of the three assays employed, when mutation carriers and non-carriers were compared. This suggests that the comet assay, micronucleus test and *γ*-H2AX nuclear staining are not capable of distinguishing between *BRCA1* mutation carriers and non-carriers.

Deficient DNA repair may not be a phenotype displayed by heterozygous *BRCA1* mutation carriers; that is, the presence of one functional allele among mutation carriers may be sufficient to maintain DNA repair at adequate levels. Alternatively, there may in fact be a phenotype associated with the BRCA1 heterozygote state, but this panel of assays is not be capable of discriminating between women with and without a *BRCA1* gene mutation. We chose not to measure bleomycin-induced chromosome aberrations because this assay is labour intensive and we hoped to identify a less costly biomarker. Furthermore, we studied relatively small numbers of cases and controls (25 of each) and our power was limited to detecting large effects. We only evaluated DNA repair at one radiation dose level and at one time period post-irradiation. It is possible that we would have better discrimination if we were to study DNA repair efficiency at various doses and over a longer-time period. However, it should be noted that each of the three assays was highly sensitive for detecting the effect of radiation *per se*; that is, post-irradiation levels of DNA damage were clearly elevated above baseline.

Kowalska *et al* (2005) reported that carriers of a *BRCA1* mutation had a significantly greater frequency of bleomycin-induced chromosome breaks then non-carrier relatives. In a recent publication from the UK, the mean number of chromosomal aberrations (translocations and breaks) per metaphase cell were scored at 24 h, and 6 days following high-dose irradiation (8 Gy). After 6 days, chromosomal damage was found to be significantly higher in lymphocytes from heterozygous *BRCA1* mutation carriers compared with normal controls (average number of aberrations per mitosis was 3.5 for cases and 1.6 for controls, *P*=0.0001) (Kote-Jarai *et al*, 2006). No difference in aberrations was detected at 24 h post-irradiation. The authors concluded that lymphocytes heterozygous for *BRCA1* demonstrate an impaired capacity to efficiently repair DNA damage following irradiation resulting in the continued existence of cells with chromosomal aberrations. This group also reported differential gene expression in normal breast fibroblasts after radiation-induced DNA damage in carriers *vs* controls (Kote-Jarai *et al*, 2004). Among the genes that were differentially expressed, included the downregulation of *RAD51*, which encodes a protein known to interact with BRCA1 and participates in the repair of DNA double strand breaks (Welcsh *et al*, 2000).

Other groups have evaluated mutagen sensitivity in peripheral blood lymphocytes, fibroblasts, and lymphoblastoid cell lines, from women with *BRCA1* mutations. Results have been conflicting (reviewed in Speit and Trenz (2004)). Speit *et al* (2000) reported that lymphocytes from women carrying a heterozygous *BRCA1* mutation show enhanced sensitivity to *γ*-irradiation, as assessed by the micronucleus test, but not the comet assay (Rothfuss *et al*, 2000; Speit *et al*, 2000; Trenz *et al*, 2002, 2003b). A second group showed that carriers of a *BRCA1* or *BRCA2* mutation were similar to controls with respect to their capacity to rejoin X-ray induced DNA breaks, assessed by pulsed-field electrophoresis in fibroblasts and the comet assay in lymphocytes (Nieuwenhuis *et al*, 2002). In another study, lymphocytes from *BRCA1* and *BRCA2* mutation carriers showed enhanced sensitivity to radiation by measuring chromatid breaks (Buchholz *et al*, 2002). In a small study of nine individuals with germ-line *BRCA1* mutations, Baria *et al* (2001) found that the micronucleus test (also referred to as the G_0_ micronucleus test) was useful at discriminating between carriers and non-carriers. Similarly, Baeyens *et al* (2002) observed enhanced radiosensitivity among breast cancer patients with a *BRCA1* or *BRCA2* mutation, *vs* a control group of healthy women, with the micronucleus test but not the G_2_ assay (number of chromatid breaks). It is interesting to note that for the micronucleus test, radiation was provided at both a low and high dose rate, and that enhanced sensitivity among women with a mutation was documented for the low dose rate (78 *vs* 33% for low and high dose rate, respectively). Others have suggested that radiation applied at a low dose rate allows better discrimination between radiosensitive and non-sensitive individuals (Jones *et al*, 1995).

Earlier studies employing lymphoblastoid cell lines with *BRCA1* (or *BRCA2*) mutations demonstrated greater sensitivity to the chromosome damaging effects of *γ-*radiation, and of hydrogen peroxide, compared to cells from healthy controls (as assessed by the micronucleus test or the radiation-induced chromatid break assay) (Foray *et al*, 1999; Speit *et al*, 2000). Recently, Trenz *et al* (2003a, 2005) demonstrated no difference in mutagen sensitivity using lymphoblastoid cell lines from women with and without a heterozygous *BRCA1* mutation, thus highlighting the limitations of using cell lines to evaluate mutagen sensitivity and DNA repair. Nieuwenhuis *et al* (2002) found no difference in the capacity of heterozygous *BRCA1* or *BRCA2* mutation carriers to rejoin radiation-induced DNA breaks in fibroblasts; whereas, dermal fibroblasts from *BRCA1* and *BRCA2* mutation carriers showed enhanced radiosensitivity as assessed by the *in vitro* radiation clonogenic survival assay (Buchholz *et al*, 2002).

In the current study, we used three assays to quantify DNA damage and subsequent repair in peripheral blood lymphocytes taken from our study population before and following exposure to *γ*-irradiation. This provided us with a measure of DNA repair kinetics rather than an assessment of the fidelity of repair. Other investigators that have chosen to evaluate the fidelity rather than the speed of DNA double-strand break repair in lymphoblastoid cell lines have reported mixed results (Baldeyron *et al*, 2002; Coupier *et al*, 2004). Using the host cell end-joining assay, Baldeyron *et al* (2002) observed that cell lines heterozygous for *BRCA1* (with truncating mutations) had reduced fidelity of DNA double-strand break repair by DNA end-joining compared with the control cell lines; similarly, Coupier *et al* (2004) reported that fidelity of end-joining was impaired in four cell lines harbouring *BRCA1* missense mutations. These findings suggest that specific *BRCA1* mutations may affect the efficiency rather than the speed of DNA repair. The results from the comet assay and *γ*-H2AX staining illustrate that there was no difference in the repair kinetics in our study population. Whereas we did not detect any differences with the MNT, which provides a measure of fidelity.

Depending on the type of DNA lesion, the appropriate repair pathway will be activated (Hoeijmakers, 2001; Kastan and Bartek, 2004). The BRCA1 protein is involved in double-strand break repair, but also in other repair pathways, including transcription-coupled repair, nucleotide-excision repair and mismatch repair (Trenz *et al*, 2003b). As both carriers and non-carriers showed similar sensitivities to radiation-induced damage, perhaps quantifying other forms of damage and subsequent repair (i.e. at the chromosomal level) may be more effective in discriminating between carriers and non-carriers.

We did not observe any difference in radiation sensitivity among our sample of women using the panel of three assays. Subsequently, they are unlikely to be predictors of breast cancer susceptibility. At the present time, there is no standard functional test that can be applied routinely to allow discrimination between carriers and non-carriers. Even though various studies described above have detected differences in mutagen sensitivity, they were often limited by their sample size, choice of study population (i.e. some included both men and women, cancer patients), and lymphoblastoid cell lines that may display a different response to radiation when compared to peripheral blood lymphocytes (Baeyens *et al*, 2004). Strengths of our study include the evaluation of multiple endpoints (before and after exposure to *γ*-irradiation), a relatively large sample of carriers, the use of controls who were from a family with a previously identified mutation, and employing a single technician who was blinded to the mutation status of the samples. Nontheless, these findings collectively suggest that perhaps markers of chromosomal damage (i.e. chromosomal breaks, chromatid breaks) may serve as better biomarkers of risk. Although there are numerous techniques to assess DNA repair capacity, there is presently no gold standard (Berwick and Vineis, 2000; Hemminki and Thilly, 2004).

Various cytogenetic end-points, including counting chromosomal aberrations, sister chromatic exchanges and micronuclei have previously been utilised as biomarkers of cancer susceptibility in non-carriers (reviewed in Norppa (2004)). Epidemiological evidence supports a predictive value of an elevated frequency of chromosomal aberrations in peripheral blood lymphocytes (Hagmar *et al*, 2004). In the Nordic (Hagmar *et al*, 1994), Italian (Bonassi *et al*, 1995), and Czech (Smerhovsky *et al*, 2001), cohort studies, the authors evaluated the association between the frequency of chromosomal aberrations, sister chromatid exchange or micronuclei in peripheral blood lymphocytes of individuals and the subsequent risk of cancer. The reported findings showed an approximately two-fold increase in the risk of cancer among those with the highest frequencies of chromosomal aberrations (reviewed in Hagmar *et al* (2004)). No association was found with the other markers (i.e. sister chromatid exchanges or micronuclei). Collectively, these studies have demonstrated that chromosomal aberrations can predict cancer risk in humans in some settings.

The advantages and disadvantages of using DNA repair as a marker of cancer susceptibility has received considerable attention (Berwick and Vineis, 2000). Limitations include inter- and intra-individual variability in the assay, test reliability, and biological plausibility (reviewed in Berwick and Vineis (2000)). An underlying assumption in using these assays is that DNA repair capacity in peripheral lymphocytes is representative of repair in the breast tissue. It is possible that repair capacity differs between breast tissue and lymphocytes; however, BRCA1 is expressed in most tissues and cell types, especially during the S and G2 phases (reviewed in Venkitaraman (2002)). It is possible that the dose of radiation used in the present study may have been too high to detect a difference. However, others have successfully detected differences with 4 Gy min^−1^ (Jones *et al*, 1995).

## CONCLUSION

Based on the functions of the BRCA1 protein in DNA repair and the prevention of oxidative stress, it has been proposed that there is potential for the prevention of hereditary breast cancer through lifestyle modification, including dietary changes that decrease oxidative DNA damage or enhance DNA damage repair pathways (Kotsopoulos and Narod, 2005). We did not find any significant differences in DNA repair capacity in peripheral blood lymphocytes between *BRCA1* mutation carriers and non-carrier controls using the three different measures. The comet assay, micronucleus test, and *γ*-H2AX staining are not substitutes for other cytogenetic markers (i.e. the frequency of chromosome breaks) as markers of cancer susceptibility in this high-risk group. The ability of individual genetic and non--genetic factors to predict DNA repair capacity requires further evaluation.

## Figures and Tables

**Table 1:** Principal characteristics of the study participants, by mutation status

**Variable**	***BRCA1* mutation carriers (*BRCA1^+/−^*) *N*=25**	**Non-carriers (*BRCA1^WT^*) *N*=25**	***P* a**
Age at interview (years), mean (s.d.)^b^	43.56 (9.81)	44.62 (11.19)	0.738
Age at menarche, mean (s.d.)	12.31 (1.40)	12.36 (1.70)	0.916
Age at first birth, mean (s.d.)^c^	26.50 (5.00)	26.79 (4.80)	0.873
Height (inches), mean (s.d.)	64.38 (2.52)	64.02 (2.15)	0.589
Weight (pounds), mean (s.d.)	147.84 (30.46)	154.04 (34.77)	0.506
BMI (kg m^−2^), mean (s.d.)	25.02 (4.70)	26.36 (5.63)	0.367
			
*Menopausal status, n (%)*			
Premenopausal	10 (40)	17 (68)	
Postmenopausal	15 (60)	8 (32)	0.047
			
*Hormone replacement therapy use* d *, n (%)*			
Non-user	21 (84)	25 (100)	
User	4 (16)	0 (0)	0.037
			
*Oral contraceptive use* d *, n (%)*			
Non-user	23 (92)	20 (80)	
User	2 (8)	5 (20)	0.221
			
*Smoking status* d *, n (%)*			
Non-user	25 (100)	22 (88)	
User	0 (0)	3 (12)	0.074
			
Total alcoholic drinks per day, mean (s.d.)	0.73 (1.57)	0.70 (0.69)	0.935
			
Energy intake (kcal day^−1^), mean (s.d.)	1730.09 (540.23)	1718.02 (443.41)	0.934
			
Total hours of physical activity per week, mean (s.d.)	19.03 (7.25)	22.60 (5.17)	0.065

a All *P*-values are univariate and were derived using the Student's *t*-test for continuous variables and the chi-square test for categorical variables.

b s.d.=standard deviation.

c Among parous women.

d Current use.

**Table 2:** Crude and adjusted mean tail moments in study population, stratified by *BRCA1* mutation status

		**Crude mean (±s.e.m.)**			**Adjusted mean^b^ (±s.e.m.)**		
**Dose (Gy)**	**Time after irradiation (h)**	***BRCA1^+/−^***	***BRCA1^WT^***	***P* a**	***BRCA1^+/−^***	***BRCA1^WT^***	***P* a**
0	0	2.63 (0.31)	2.80 (0.31)	0.696	2.99 (0.31)	2.74 (0.15)	0.472
2	0	7.99 (0.67)	7.96 (0.66)	0.974	8.37 (0.59)	7.90 (0.32)	0.486
2	1	3.64 (0.44)	3.71 (0.38)	0.907	4.05 (0.41)	3.68 (0.17)	0.396

a The Student's *t*-test used to test for differences in the crude and adjusted means of the tail moments between *BRCA1* mutation carriers and non-carriers.

b Multivariate linear regression included terms for age (years), BMI (kg m^−2^), current smoker (yes/no), alcohol intake (total drinks of per day), physical activity (total hours of per week), and total daily caloric intake (kcal).

**Table 3:** Crude and adjusted mean micronucleus frequencies, stratified by *BRCA1* mutation carriers and non-carriers

		**Crude mean (±s.e.m.)**			**Adjusted mean^b^ (±s.e.m.)**		
**Dose (Gy)**	**Time after irradiation (h)**	***BRCA1^+/−^***	***BRCA1^WT^***	***P* a**	***BRCA1^+/−^***	***BRCA1^WT^***	***P* a**
0	0	25.06 (3.39)	18.00 (2.89)	0.120	20.89 (3.26)	19.96 (1.81)	0.794
2	0	132.77 (12.39)	128.84 (12.03)	0.821	124.70 (4.69)	139.79 (7.37)	0.114

a The Student's *t*-test used to test for differences in the crude and adjusted means of the tail moments between *BRCA1* mutation carriers and non-carriers.

b Multivariate linear regression included terms for age (years), BMI (kg m^−2^), current smoker (yes/no), alcohol intake (total drinks of per day), physical activity (total hours per week), and total daily caloric intake (kcal).

**Table 4:** Crude and adjusted mean *γ*-H2AX foci in study population, stratified by *BRCA1* mutation status

		**Crude mean (±s.e.m.)**			**Adjusted mean^b^ (±s.e.m.)**		
**Dose (Gy)**	**Time after irradiation**	***BRCA1^+/−^***	***BRCA1^WT^***	***P* a**	***BRCA1^+/−^***	***BRCA1^WT^***	***P* a**
0	0 h	11.74 (4.63)	8.78 (3.03)	0.621	9.46 (4.23)	11.67 (1.77)	0.641
2	30 min	30.14 (4.50)	25.68 (2.93)	0.447	25.00 (5.31)	31.18 (2.47)	0.323
2	3 h	31.17 (4.79)	32.31 (4.39)	0.864	25.77 (4.89)	33.24 (3.68)	0.229

a The Student's *t*-test used to test for differences in the crude and adjusted means of the tail moments between *BRCA1* mutation carriers and non-carriers.

b Multivariate linear regression included terms for age (years), BMI (kg m^−2^), current smoker (yes/no), alcohol intake (total drinks of per day), physical activity (total hours per week), and total daily caloric intake (kcal).
